# Revision of Poliaspis (Hemiptera, Coccoidea, Diaspididae), with descriptions of 8 new species from Australia

**DOI:** 10.3897/zookeys.137.1786

**Published:** 2011-10-14

**Authors:** Nate Hardy, Rosa C. Henderson

**Affiliations:** 1 Entomology, Department of Biology, University of New Mexico, Museum of Southwestern Biology, 167 Castetter Hall, MSC03 2020, Albuquerque, NM 87131–0001; 2 Landcare Research, Private Bag 92170, Auckland 1142, New Zealand

**Keywords:** taxonomy, species descriptions, armored scale insects

## Abstract

Eight new Australian species of *Poliaspis *are described and illustrated: *Poliaspis alluvia* **sp. n.**, *Poliaspis araucariae* **sp. n.**, *Poliaspis ceraflora* **sp. n.**, *Poliaspis naamba* **sp. n.**, *Poliaspis nalbo* **sp. n.**, *Poliaspis narungga ***sp. n.**, *Poliaspis ozothamnae* **sp. n.**, and *Poliaspis waibenensis* **sp. n.** Two described species are transferred into *Poliaspis* and are redescribed and illustrated: *Lineaspis callitris* (Laing) originally described by Laing as a species of *Poliaspis*, is transferred back into *Poliaspis* as *Poliaspis callitris* Laing, **comb. rev.**, and *Leonardaspis wilga* (Leonardi) is transferred to *Poliaspis* as *Poliaspis wilga* (Leonardi), **comb. n.** Descriptions and illustrations are also provided for six of the fourteen previously-named *Poliaspis* species, including five from Australia: *Poliaspis attenuata* Brimblecombe, *Poliaspis elongata* Brimblecombe, *Poliaspis exocarpi* Maskell, *Poliaspis nitens* Fuller, and *Poliaspis syringae* Laing. Both *Poliaspis cycadis* Comstock and *Poliaspis gaultheriae* Green become junior synonyms of *Poliaspis media* Maskell. The species not treated here are *Poliaspis intermedia* Fuller (the location of the types is unknown and Fuller’s description is inadequate), *Poliaspis casuarinicola* Lindinger (missing types), *Poliaspis incisa *Takagi and de Faveri (recently, and well described in Takagi and de Faveri 2011), and the six New Zealand species recently revised by Henderson (2011). In addition, *Laingaspis lanigera* (Laing), the adult female of which has 8 clusters of perivulvar pores – as in *Poliaspis* species – is redescribed and illustrated. Lectotypes are designated for *Laingaspis lanigera*, *Poliaspis callitris*, *Poliaspis exocarpi*, *Poliaspis media*, and *Poliaspis wilga*. A key is provided to the species of *Poliaspis*, excluding *Poliaspis casuarinicola *and *Poliaspis intermedia* butincluding *Poliaspis incisa* and the New Zealand species: *Poliaspis chathamica* Henderson, *Poliaspis floccosa* Henderson, *Poliaspis lactea* (Maskell), *Poliaspis media* Maskell, *Poliaspis raouliae* Henderson and *Poliaspis salicornicola* Henderson.

## Introduction

Many armored scale insects (Diaspididae) are pests, and armored scales are disproportionately common in invasive faunas. About 2,500 species of armored scale insects have been described, and ten percent of these (250 spp.) are known to occur in Australia (http://scalenet.info/country_taxon/Australia/Diaspididae/).

Maskell (1880) erected the genus *Poliaspis* by monotypy for *Poliaspis media*, a New Zealand species having eight groups of perivulvar pores occurring on the ventromedial surfaces of abdominal segments 6 and 5. Fourteen additional species sharing that distinction were described and added to *Poliaspis* by Maskell and other authors (Comstock 1883; Maskell 1892; Fuller 1897, 1899; Lidgett 1898; Laing 1929; Brimblecombe 1959; Henderson 2011). Seven described species of *Poliaspis* are from Australia. The Australian Plant Pest Database, integrating specimen data from several Australian insect collections, contains 398 sample records for *Poliaspis* species. Only 25% of these are identified to species. Amongst the unidentified material in the Australian National Insect Collection, and the Queensland Primary Industries Insect Collection, eight undescribed *Poliaspis *species were recognized.

Here, these eight new species of *Poliaspis* as well as six of the fourteen previously-named species are described and illustrated. We transfer two described species into *Poliaspis* and redescribe and illustrate these. We also redescribe and illustrate *Laingaspis lanigera* (Laing), the adult female of which has a similar distribution of perivulvar pores, but differs substantially in another key morphological feature. A key to the species of *Poliaspis* is provided, excluding *Poliaspis casuarinicola* and *Poliaspis intermedia*, but including *Poliaspis incisa* Takagi & De Faveri, recently described from Northern Queensland on mangroves (Takagi and De Faveri 2011).

## Methods

Depositories are abbreviated as follows: ASCU, Agricultural Scientific Collections Unit, Orange Agricultural Institute, New South Wales; BMNH, the Natural History Museum, London, UK; NZAC, New Zealand Arthropod Collection, Auckland, NZ; QDPI, Queensland Primary Industries and Fisheries, Brisbane, Queensland, Australia; QMBA, Queensland Museum, Brisbane.

Measurements were made using the measurement tools in NIS-Elements BR 3.00, SPI (Build 455). For species with ≤ 10 specimens, measurements were taken from all specimens; for those with >10 specimens, 10 specimens were measured, chosen to represent the range of host plants and geographic localities present in the sample. The morphological terms for Diaspididae follow those of Miller and Davidson (2005). To make a clear distinction between the gland spines occurring on the pygidial margin, and the gland spines / tubercles occurring in submarginal areas anterior of the pygidium, all gland spines / tubercles anterior of the pygidium are referred to as gland tubercles. Because scale insects are bilaterally symmetrical, only one side of the body is described unless discussing features on the midline. For example, the number of anterolateral perivulvar pores given is that on one side of the body (not the sum of both sides), but the number of anteromedial pores is the total found in the cluster of pores extending across the midline (rather than dividing that number by 2). Species descriptions inherit and override attributes from the generic description, which can be thought of as an abstract base class as per the recommendations of Cook et al. (2010).

Following the convention for scale insects, each figure displays the dorsal body surface on the left side of the page, and the ventral body surface on the right. Enlargements of diagnostic features are located around the margin of each main figure. Geographic coordinates are provided for each collection location (with a few exceptions). If this information was not part of the original collection data (most cases), approximated coordinates are provided in square brackets. We estimated coordinates via the Google Geocoding API (http://code.google.com/apis/maps/documentation/geocoding/), automating requests via a Python script (available from NBH by request).

## Taxonomy

### Genus Poliaspis

Maskell

http://species-id.net/wiki/Poliaspis

Poliaspis Maskell, 1880: 293. Type species: *Poliaspis media* Maskell, monotypy.

#### Description.

*Scale cover.* Round to elongate-oval, white, flocculent wax sometimes present, exuvia terminal (after Henderson 2011).

*Slide-mounted adult female*. Body outline variable: linear, turbinate, pyriform, fusiform or oval, prepygidial abdominal margin weakly incised between segments to strongly lobed. Margin of pygidium rounded; incised between median lobes in some species, not incised in others. Two pairs of lobes in all species except *Poliaspis wilga* comb. n. (only medial pair) and some New Zealand species (3^rd^ lobe represented by three pointed projections); median lobes zygotic (except in *Poliaspis ceraflora*), parallel or divergent, apex variable – rounded or pointed; pair of setae between median lobes in most species; second lobes bi-lobed or undivided; basal scleroses present or absent. Simple gland spines present; most species with 1 gland spine on each side of each pygidial segment (other than segment 8), but gland spines may be absent on segment 7 (in area adjacent to lateral margin of medial lobe, e.g. *Poliaspis ozothamnae* sp. n.), or absent from pygidial segment 5 (*Poliaspis ceraflora* sp. n., *Poliaspis callitris* comb. rev.), or 2–6 may be present on each side of segments 5 and 6 (*Poliaspis ozothamnae*; *Poliaspis nalbo* sp. n.); length of gland spines variable, from about as long as median lobes to > 5 × length of median lobes. Anus in anterior third of pygidium; opening round. Trilocular pores in cluster near each anterior spiracle, some species also with pores near posterior spiracles. Antenna with 1 or 2 fleshy setae. Perivulvar pores quinquelocular, in 8 groups; 5 groups on abdominal segment 6, and 3 groups on abdominal segment 5. Dorsal ducts 2-barred; ducts on pygidial margin larger than medial ducts in most species; distribution of enlarged marginal ducts: 1 between median and second lobes; 1–2 on segment 6, laterad of second lobes; 2 on segment 5; dorsal ducts (other than those on margin) decreasing in size anteriorly; absent from abdominal segment 7; discrete submarginal and submedial rows of ducts present on any of abdominal segments 2–6: 1–10 submedial ducts present on abdominal segment 6, 4–12 submarginal and 4–15 submedial ducts present on segment 5. Some species with dorsal boss present on submargin of each of abdominal segments 1 and 3. Small ducts similar to dorsal ducts present on ventral submargin. Ventral gland tubercles in marginal / submarginal clusters on thoracic and pre-pygidial abdominal segments. Microducts present on venter, at least along abdominal submargin.

#### Comments.

Nearly all other armored scale insect species have perivulvar pores in no more than 5 clusters, and restricted to abdominal segment 7. Species of *Leucaspis* Signoret and *Lopholeucaspis* Balachowsky are exceptions to this generalization; more than 5 clusters of multilocular pores may be present on the abdomen, but the extra pores occur on the submargin of abdominal segments 6 and 5. More pertinent exceptions are species in the African genera *Rolaspis* Hall, *Tecaspis* Hall, and *Dentachionaspis* MacGillivray, which have extra perivulvar pores, which occur in the same places as in *Poliaspis* (Hall 1946; Munting 1965, 1967). Described African species with extra groups of perivulvar pores invariably have marginal macroducts with elongate ductules. This feature is enough of a reason for us to refrain from taking any nomenclatural action at this time.

#### Key to species of *Poliaspis* (excluding *Poliaspis intermedia*, and *Poliaspis casuarinicola*)

* denotes New Zealand species

**Table:** 

1	Gland spines either absent or more than one gland spine on each side of at least some pygidial segments	2
–	One gland spine on each pygidial segment	7
2	Gland spines absent; marginal macroducts not differentiated from dorsal macroducts; dorsal ducts on pygidium numerous, not arranged into discrete submedial and submarginal clusters; medial lobe pointed, smaller than second lobe	*Poliaspis narungga* sp. n.
–	Gland spines present; marginal macroducts usually larger than dorsal ducts; dorsal ducts on pygidium arranged into distinct, transverse submedial and submarginal clusters; medial lobe either larger or smaller than second lobe	3
3	Median lobes non-zygotic	*Poliaspis ceraflora* sp. n.
–	Median lobes zygotic	4
4	One gland spine on each side of each of abdominal segments 7 and 6; gland spines absent from segment 5; median lobes much smaller than second lobes	*Poliaspis callitris* comb. rev.
–	Two or more gland spines present on each side of each of abdominal segments 6 and 5, or on 5 and 4; median lobes prominent, much larger than second lobes	5
5	Marginal macroducts not much larger than dorsal ducts on pygidium, 1 present on each side of abdominal segment 7, and in a discrete cluster of 2–5 ducts on segment 6	*Poliaspis chathamica* Henderson*
–	Marginal macroducts clearly larger than dorsal ducts on pygidium, 1 present on each side of abdominal segment 7, and 0 or 1 on segment 6	6
6	Gland spine present between medial and second lobes; cluster of gland tubercles anterior to anterior spiracles	*Poliaspis nalbo* sp. n.
–	Gland spine absent between medial and second lobes; no cluster of gland tubercles anterior to anterior spiracles	*Poliaspis ozothamnae* sp. n.
7	Body shape linear	8
–	Body shape fusiform, pyriform, or oval, not linear	9
8	Median lobes longer than wide	*Poliaspis attenuata* Brimblecombe
–	Median lobes wider than long	*Poliaspis elongata* Brimblecombe
9	Second lobes absent	*Poliaspis wilga*(Leonardi), comb. n.
–	Second lobes present	10
10	Median lobes smaller than second lobes	11
–	Median lobes larger than or equal to second lobes	14
11	Numerous marginal macroducts crowded along the pygidial margin and submargin, without a clear gap between those on each pygidial segment	*Poliaspis floccosa* Henderson*
–	No more than 2 enlarged macroducts on margin of each pygidial segment, with a clear gap between those on each pygidial segment	12
12	About 7 submedial ducts on each side of abdominal segment 6; median lobes short and broad, pygidial margin between lobes not incised	*Poliaspis nitens* Fuller
–	≤ Five submedial ducts on each side of abdominal segment 6; median lobes not short and broad, pygidial margin between lobes incised	13
13	Median lobes divergent, each lobe with rounded apex; 1–2 submedial duct on dorsal surface of abdominal segment 6; gland spines ca. 2–3 × as long as median lobes	*Poliaspis araucariae* sp. n.
–	Median lobes parallel, each lobe with pointed or notched apex; 2–5 ducts on dorsal surface of abdominal segment 6; gland spines ca. 4–5 × as long as median lobes	*Poliaspis exocarpi* Maskell
14	Median lobes prominent, with more than half total lobe length extending beyond margin	15
–	Much less than half total lobe length of median lobes extending beyond margin	17
15	One enlarged marginal macroduct on each side of abdominal segment 6; lobules of lobe 2 fused into a single triangular-shaped lobe	*salicornicola* Henderson*
–	Two marginal macroducts on each side of abdominal segment 6; second lobe bi-lobed	16
16	Median lobe zygosis a broad band, posterior spiracles with 2–35 pores, anterior spiracles with 8–150 pores	*Poliaspis lactea* (Maskell)*
–	Median lobe zygosis a narrow strap, posterior spiracles with 1–3 pores, anterior spiracles with 5–19 pores	*Poliaspis syringae* Laing
17	Submedial ducts on dorsum of abdominal segment 5 in cluster 2–3 ducts deep	18
–	Submedial ducts on dorsum of abdominal segment 5 in transverse linear row	19
18	Submedial ducts present on dorsum of abdominal segment 2; ca. 8 submedial ducts on dorsum of abdominal segment 6; median lobes divergent	*Poliaspis alluvia* sp. n.
–	Submedial ducts absent on dorsum of abdominal segment 2; ca. 4 submedial ducts on dorsum of abdominal segment 6; median lobes parallel	*Poliaspis incisa* Takagi and de Faveri
19	Second lobes without basal scleroses; marginal macroducts in a group of 2–4 each side of median lobes and a group of 3–6 on each side of segment 6	*Poliaspis raouliae* Henderson*
–	Second lobes with basal scleroses; 1 marginal macroduct each side of median lobes and 2 marginal macroducts on each side of segment 6	20
20	Gland spines ca. 1 × length of marginal macroducts	*Poliaspis media* (Maskell)*
–	Gland spines ca. 2–4 × length of marginal macroducts	21
21	Conspicuous duct spur, about as long as lateral lobule of second lobe present between medial and second lobe; usually 4 submedial ducts on dorsal surface of abdominal segment 6; pores usually absent from around posterior spiracle	*Poliaspis waibenensis* sp. n.
–	Conspicuous ducts spur, about as long as lateral lobule of second lobe not present between medial and second lobe; usually 2–3 submedial ducts on dorsum of abdominal segment 6; ca. 2 pores present near posterior spiracle	*Poliaspis naamba* sp. n.

### Poliaspis alluvia

Hardy & Henderson sp. n.

urn:lsid:zoobank.org:act:62654309-96F2-413D-BD3C-1DF6CFA5BCBA

http://species-id.net/wiki/Poliaspis_alluvia

Fig. 1

#### Material examined.

Holotype: 1 adult female: Australia, QLD, Mt Whitestone [-27.67, 152.16], ex Loranthaceae, 13.6.1989, M Taylor (ANIC).

#### Description, n=1.

Slide-mounted holotype female 952 μm long, body outline pyriform, thoracic and abdominal lobes weakly produced. Pygidium with 2 pairs of lobes; median lobes divergent, with dentate apex; margin between lobes incised to a variable degree; second lobe bi-lobed, each lobule with basal sclerosis, more strongly developed on medial lobule. Gland spines 24–38 μm long, 2–3 × length of median lobes, 1 gland spine on margin of each pygidial segment; pair of setae between median lobes. Dorsal ducts smaller than marginal ducts present in rows; 8 submedial ducts present on segment 6; ca. 9 submarginal and ca. 10 submedial ducts on segment 5; ducts also present on segment 2. Perivulvar pores numerous: ca. 12 posteromedial, ca. 20 posterolateral, ca. 40 posterior, ca. 12 anteromedial, and ca. 5 anterolateral. Trilocular pores in cluster of 7–8 near anterior spiracle; 4 near posterior spiracle. Microducts few to numerous on dorsum of head, scattered anterior to anterior spiracles and mesad of gland tubercles on thorax and abdomen, few or absent on median abdomen. Antenna with 2 fleshy setae.

#### Comments.

The relatively large number (ca 8) of submedial ducts on abdominal segment 6, as well as the large number of perivulvar pores (ca 90) can be used to distinguish *Poliaspis alluvia* from other species of *Poliaspis*.

#### Etymology.

The species name is taken from the Latin word *alluvio* ,meaning flood, in commemoration of the flooding of the Brisbane river in January 2011.

**Figure 1.**
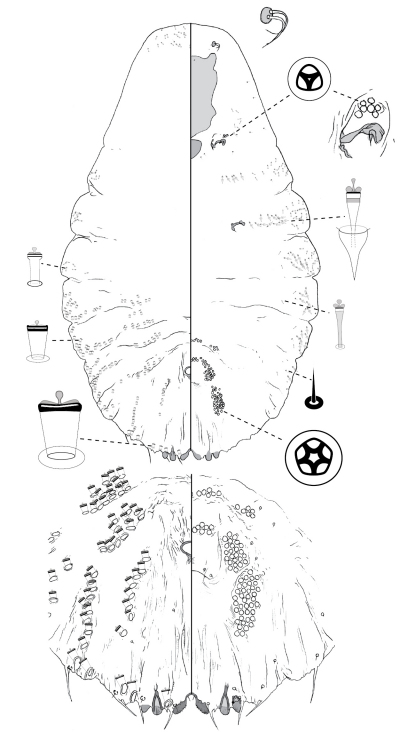
*Poliaspis alluvia* Hardy and Henderson sp. n., adult female.

### Poliaspis araucariae

Hardy & Henderson sp. n.

urn:lsid:zoobank.org:act:6A0B48DA-1DC6-4F89-8429-506E76B6EE00

http://species-id.net/wiki/Poliaspis_araucariae

Fig. 2

#### Material examined.

Holotype: female: Australia, QLD, Taromeo [-26.83, 152.12], *Araucaria bidwillii*, 1.9.1937, A Brimblecombe, 165; 1185/10387 (ANIC).

Paratypes: QLD: 10 adult females: Gallangowan [-27.93, 151.67], ex *Araucaria cunninghamii*, 15.2.1944, Se/1945 (QDPI); 8 adult females: same data as holotype (QDPI).

#### Description, n=7.

Slide-mounted adult female 724–1658 μm long (holotype 1016 μm long), body outline fusiform-pyriform, thoracic and abdominal lobes produced. Pygidium with 2 pairs of lobes; median lobes divergent, connected medially by narrow sclerotic strap, lobes with rounded apex; margin between lobes weakly incised; second lobe bi-lobed, each lobule with basal sclerosis, more strongly developed on medial lobule. Gland spines 12–27 μm long, 2–3 × length of median lobes, 1 gland spine on margin of each pygidial segment; pair of minute setae absent between median lobes. Dorsal ducts similar in size to marginal ducts; present in rows; 1–2 submedial ducts present on segment 6; ca. 6 submarginal and ca. 5 submedial ducts on segment 5. Perivulvar pores: 5–14 posteromedial, 9–20 posterolateral, 23–27 posterior, 1–8 anteromedial, and 7–12 anterolateral. Trilocular pores in cluster of 2–4 near anterior spiracle; absent from posterior spiracle. Microducts scattered on head dorsum, anterior to anterior spiracles and mesad of gland tubercles on thorax and abdomen. Antenna with 2 fleshy setae.

#### Comments.

The one or two submedial ducts on abdominal segment 6, in addition to the absence of setae between the median lobes (also absent in *Poliaspis callitris*, and *Poliaspis nitens*) can be used to distinguish *Poliaspis araucariae* from other species of *Poliaspis*.

#### Etymology.

The species name refers to the host species: *Araucaria cunninghamii* and *Araucaria bidwillii*.

**Figure 2.**
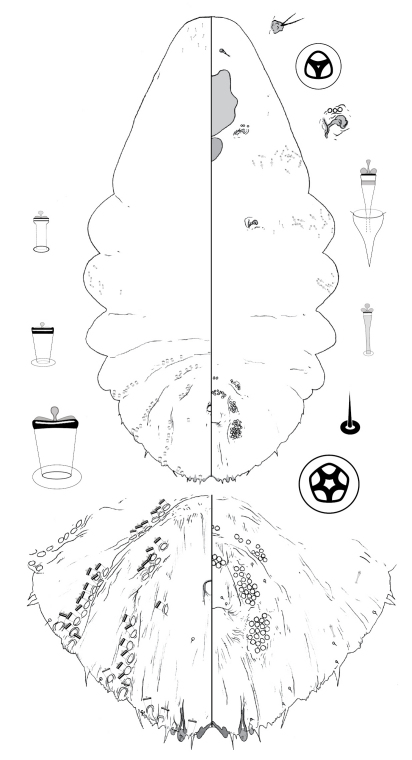
*Poliaspis araucariae* Hardy and Henderson sp. n., adult female.

### Poliaspis attenuata

Brimblecombe

http://species-id.net/wiki/Poliaspis_attenuata

Fig. 3

Poliaspis attenuata Brimblecombe 1959: 401–403

#### Material examined.

Paratype: QLD. 1 adult female: Yarraman [-26.84, 151.98], ex *Croton insularis*, 1.9.1948, A Brimblecombe (QDPI).

#### Description, n=1.

Slide-mounted paratype female 1644 μm long, body outline linear, abdominal lobes weakly produced. Pygidium with 2 pairs of lobes; median lobes divergent, longer than wide, connected medially by narrow sclerotic strap, each lobe with dentate apex; margin between lobes incised; second lobe bi-lobed, medial lobule with basal sclerosis. Gland spines 18–37 μm long, ca. 2 × length of median lobes, 1 gland spine on margin of each pygidial segment; pair of setae present between median lobes. Dorsal ducts smaller than marginal ducts; present in rows; 2 submedial ducts present on segment 6; 4 submarginal and 5–6 submedial ducts on segment 5. Perivulvar pores: 4 posteromedial, 9–10 posterolateral, 17–18 posterior, 6 anteromedial, and 3–4 anterolateral. Trilocular pores in cluster of ca. 2 near anterior spiracle; absent from posterior spiracle. Microducts scattered on dorsal surface of head, plus medial and submarginal areas of anterior abdominal segments. Antenna with 1 fleshy setae.

#### Comments.

Adult females of *Poliaspis attenuata* are most similar to those of *Poliaspis elongata* Brimblecombe. Both have elongate, linear bodies. *Poliaspis attenuata* females can be distinguished on the basis of the longer-than wide, divergent median lobes (wider than long in *Poliaspis elongata*, with rounded apices).

**Figure 3.**
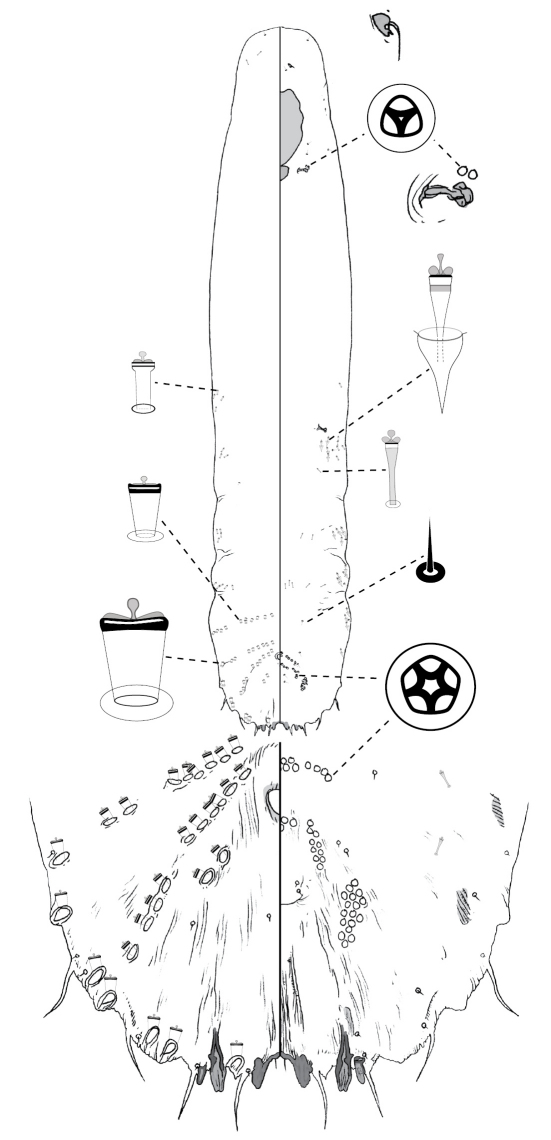
*Poliaspis attenuata* Brimblecombe, adult female.

### Poliapsis callitris

Laing comb. rev.

http://species-id.net/wiki/Poliapsis_callitris

Fig. 4

Poliaspis callitris Laing, 1929: 19–20.
Lineaspis callitris (Laing), change of combination, Borchsenius 1966: 103.

#### Material examined.

Lectotypefemale here designated, 1 of 8 specimens on slide labelled “Australia, VIC, Mallee, on *Callitris* sp., JE Dixon, no.11,1919, IBE 1385, EE Green det. *?Chionaspis striata*, *Poliaspis callitris* Laing sp. n.”The Lectotype is the only un-distorted adult female on slide. (BMNH)

Paralectotypes: (i) the remaining 7 females on the lectotype slide; (ii) 2 adult females on second slide with same collection data and no BM number (BMNH).

Other Material: QLD. 3 adult females: Australia, QLD, Lake Broadwater Conservation park [-27.35, 151.1], on stems of *Callitris* sp., 19.11.1985, J Donaldson (QDPI); 1 adult female: Southport [-27.97, 153.41], ex *Callitris columellaris*, 15.8.1953, A Brimblecombe (QDPI); 8 adult females: Southport [-27.97, 153.41], *Cupressus macrocarpa*, 10.1936, A Brimblecombe (QDPI).

#### Description, n=7.

Slide-mounted adult female 581–1188 μm long (holotype 945 μm long), body outline fusiform, without distinct thoracic and abdominal lobes. Pygidium with 2 pairs of lobes; median lobes zygotic, much smaller than medial lobule of second lobe, each lobe with pointed apex; margin between median lobes not incised; second lobe bi-lobed, lateral lobule minute in some specimens (including holotype), medial lobule with strong basal sclerosis. Gland spines 7–11 μm long, about same length as median lobes, 1 gland spine on margin of each of pygidial segments 6 and 7 (i.e. lateral of each lobe), gland spines absent from segment 5; pair of setae absent between median lobes. Dorsal ducts smaller than marginal ducts; present in rows; 2 submedial ducts present on segment 6; ca. 3 submarginal and ca. 4 submedial ducts on segment 5. Perivulvar pores: 2–5 posteromedial, 7–11 posterolateral, 5–13 posterior, 2 anteromedial, and 2–5 anterolateral. Trilocular pores in cluster of 9–15 near anterior spiracle; absent from posterior spiracle. Microducts scattered on dorsal surface of head, mesad of gland tubercles on abdomen. Antenna with 1 fleshy seta.

#### Comments.

Adult females of *Poliaspis callitris* can be distinguished from other species of *Poliaspis* on the basis of (1) setae between median lobes absent (also absent in *Poliaspis araucariae* and *Poliaspis nitens*); and (2) the relatively large number of pores near anterior spiracle (9–15) and no pores near posterior spiracle (other species of *Poliaspis* having many pores near anterior spiracle have at least a few near posterior spiracle). It shares having only 1 small gland spine on the margin of abdominal segments 6 and 7 with *Poliaspis ceraflora* , but that species has non-zygotic median lobes*.*

**Figure 4.**
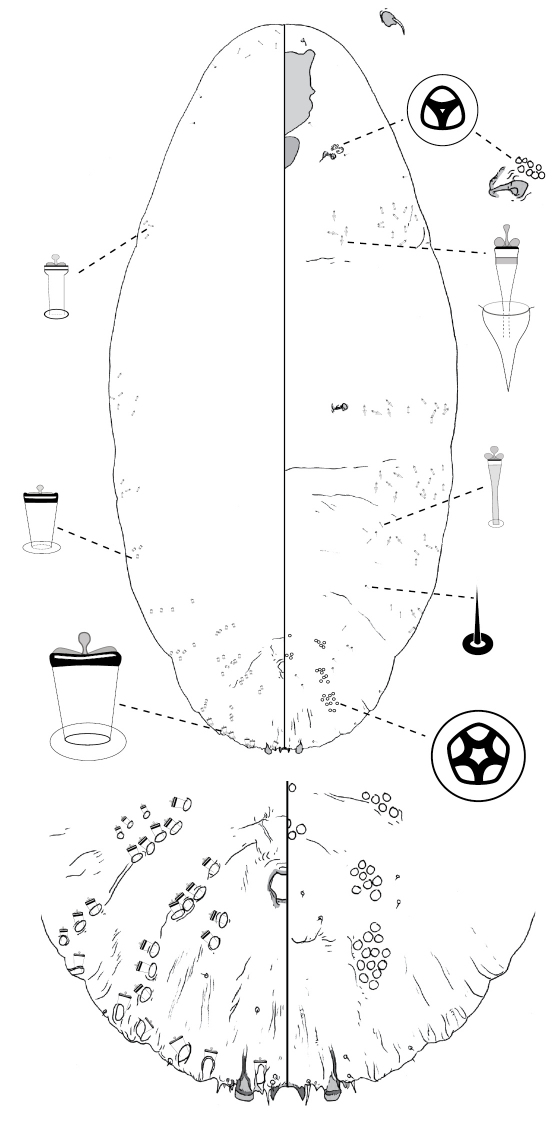
*Poliaspis callitris* Laing comb. rev., adult female.

### Poliaspis ceraflora

Hardy & Henderson sp. n.

urn:lsid:zoobank.org:act:1C15AF8B-51E2-4C01-B689-1D43E4598E3F

http://species-id.net/wiki/Poliaspis_ceraflora

Fig. 5

#### Material examined.

Holotype: female: Australia, WA, Perth [-31.95, 115.86], ex *Chamelaucium uncinatum*, 7.1989, J Donaldson (ANIC).

Paratypes: WA. 5 adult females: same data as holotype (QDPI); 6 adult females: Perth City Council Nursery, ex *Melaleuca* sp., 8.1973 (ANIC).

#### Description, n=10.

Slide-mounted adult female 807–1639 μm long (holotype 1076 μm long), body outline pyriform, thoracic and abdominal lobes weakly produced (undulate). Pygidium with 2 pairs of lobes; median lobes non-zygotic (separate), each lobe with rounded apex; margin between median lobes not incised; second lobe bi-lobed, lateral lobule minute, medial lobule with small basal sclerosis. Gland spines minute, 7–11 μm long, 1 gland spine lateral of each lobe; pair of setae absent between median lobes. Dorsal ducts smaller than marginal ducts; present in non-linear clusters; ca. 4 submedial ducts present on segment 6; ca. 6 submarginal and ca. 8 submedial ducts on segment 5. Marginal ducts: 1 on segment 7, 3 on segment 6, 3–4 on segment 5. Perivulvar pores: 4–5 posteromedial, 9–11 posterolateral, 15–24 posterior, 4–5 anteromedial, and 4–6 anterolateral. Trilocular pores in cluster of 4–5 near anterior spiracle; absent from posterior spiracle. Microducts numerous on dorsal surface of head, scattered mesad of gland tubercles on thorax, in medial and submarginal areas of abdomen. Antenna with 1 fleshy seta.

#### Comments.

This is the only species of *Poliaspis* with non-zygotic median lobes. The two pairs of minute gland spines are also distinctive, although *Poliaspis callitris* Laing shares the character of possessing only two pairs of small gland spines.

#### Etymology.

The species name is derived from the Latin words for wax (*cera*) and flower (*floris*), in reference to the common name, wax flower, of the host plant genus *Chamelaucium*.

**Figure 5.**
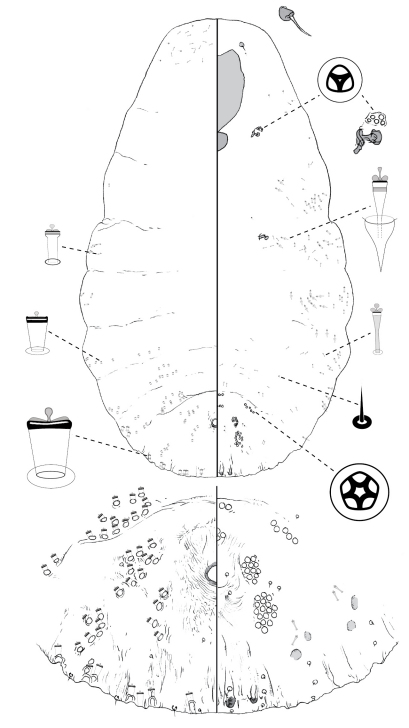
*Poliaspis ceraflora* Hardy and Henderson sp. n., adult female.

### Poliaspis elongata

Brimblecombe

http://species-id.net/wiki/Poliaspis_elongata

Fig. 6

Poliaspis elongata Brimblecombe, 1959: 403–405

#### Material examined.

Paratypes: QLD. 6 adult females: Tugun [-28.14, 153.5], ex *Leptospermum whitei*, 30.9.1947, A Brimblecombe (QDPI).

#### Description, n=6.

Slide-mounted adult female 1323–1886 μm long, body outline linear, with margins of anterior abdominal segments distinctly lobed. Pygidium with 2 pairs of lobes; median lobes zygotic, each lobe wider than long, with rounded apex; margin between lobes not deeply incised; second lobe bi-lobed, lateral lobule small, medial lobule with strong basal sclerosis. Gland spines 22–40 μm long, about 3 × length of median lobes, 1 gland spine on lateral margin of each pygidial segment; pair of minute setae between median lobes. Dorsal ducts smaller than marginal ducts; present in loose rows; 3 submedial ducts present on segment 6; ca. 4 submarginal and ca. 8 submedial ducts on segment 5. Perivulvar pores: 5–8 posteromedial, 8–12 posterolateral, 17–20 posterior, 5–8 anteromedial, and 3–7 anterolateral. Trilocular pores in cluster of 4 near anterior spiracle; absent from posterior spiracle. Microducts numerous on dorsal surface of head, scattered on ventral surface of abdomen. Antenna with 1 fleshy seta.

#### Comments.

Adult females of *Poliaspis elongata* are most similar to *Poliaspis attenuata*. See comments under *Poliaspis attenuata* for discussion.

**Figure 6.**
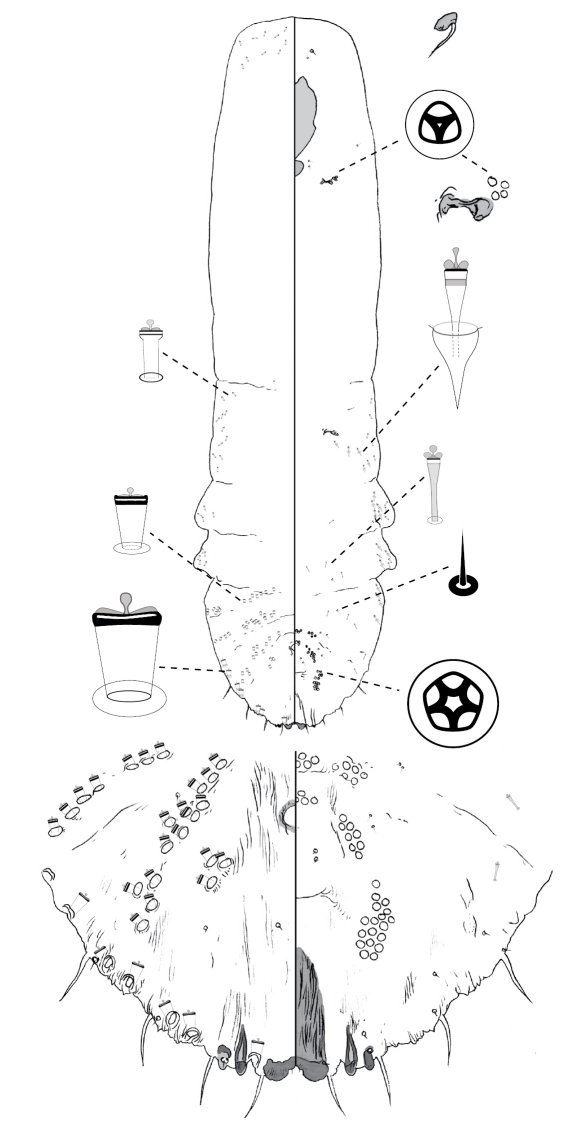
*Poliaspis elongata* Brimblecombe, adult female.

### Poliaspis exocarpi

Maskell

http://species-id.net/wiki/Poliaspis_exocarpi

Fig. 7

Poliaspis exocarpi Maskell, 1892: 17.

#### Material examined.

Lectotype: female, here designated to preserve nomenclatural stability. On an original slide labelled “*Poliaspis exocarpi*, adult female, 1891, W.M.M.”. AUSTRALIA, Mordialloc [-38.00, 145.09], near Melbourne, on *Exocarpus cupressiformis*, by Mr. French (NZAC). Paralectotypes: (i) 1 female, slide label data as above (NZAC); (ii) (not examined) 1 (BMNH); 12 (USNM).

Other material: QLD. 1 adult female: Amamoor [-26.35, 152.68], ex pumpkin, 8.12.1927, H 151a (QDPI); 1 adult female: Amamoor [-26.35, 152.68], ex *Cucurbita maxima*, 12.12.1927, H 151b (QDPI); 13 adult females: Bamaga [-10.89, 142.39], on leaves of *Asteromyrtus lysicephala*, 10.9.1983, J Donaldson (QDPI); 3 adult females: Chinchilla [-26.74, 150.63], ex *Eremocitrus glauca*, 1.12.1981, J Baker (QDPI); 9 adult females: Dalby-Tara-St George Road Junction, on stems of *Apophyllum anomalum*, 21.11.1985, J Donaldson (QDPI); 4 adult females: Dauan Island [-9.43, 142.53], on leaves, 25.6.1995, J Grimshaw, JFG 2722 (QDPI); 4 adult females: Drillham [-26.64, 149.98], on leaves of *Geijera parviflora*, 30.4.1953, A Brimblecombe (QDPI); 7 adult females: Emu Vale [-28.23, 152.25], ex *Euroschinus falcata*, 12.2.1939, A Brimblecombe, 399 (QDPI); 13 adult females: Fletcher [-28.77, 151.87], ex *Jacksonia scoparia*, 11.1949, (QDPI); 4 adult females: Gabba Island [-9.77, 142.63], on leaves of *Exocarpus latiflolius*, 13.6.2000, J Grimshaw, JFG 5107 (QDPI); 4 adult females: Gabba Island [-9.77, 142.63], on leaves of *Exocarpus latiflolius*, 4.6.2003, J Grimshaw (QDPI); 1 adult female: Hopevale [-15.29, 145.11], ex *Cycas* sp., 6.6.2000, B Waterhouse, JFG 5380 (QDPI); 10 adult females: Imbil [-26.47, 152.7], ex *Euroschinus falcata*, 10.1936 (QDPI); 7 adult females: Jandowae [-26.78, 151.11], on leaves of *Eremocitrus glauca*, 26.6.1989, J McAlpine, N5149 (QDPI); 3 adult females: Lake Broadwater Conservation park [-27.35, 151.1], on leaves of *Amyema congener*, 21.11.1985, J Donaldson (QDPI); 5 adult females: Punsand Bay [-10.87, 142.39], on leaves of *Garcinia warvenii*, 24.7.2003, J Grimshaw, (QDPI); 7 adult females: Texas [-28.85, 151.17], ex *Geijera parviflora*, 10.1954, A Brimblecombe (QDPI); 2 adult females: Yarraman [-26.84, 151.98]**,** ex *Xanthoxylon brachyacanthum*, 6.6.1947, A Brimblecombe, IIE number 1283/10665 (BMNH). VIC. 3 adult females: Dandenong Range [-37.83, 145.35]**, **ex *Exocarpus stricta*, E Green, BM Reg. Number 1926–415 (BMNH).

#### Description, n=10.

Slide-mounted adult female 847–1544 μm long, body outline pyriform to fusiform, with weakly-developed lobes on pre-pygidial abdominal segments. Pygidium with 2 pairs of lobes; median lobes zygotic, parallel, each lobe about as wide as long, with pointed apex, 1–2 notches present in some specimens; margin between median lobes weakly incised; second lobe bi-lobed, lobules similar in size, medial lobule with basal sclerosis. Gland spines 27–43 μm long, about 4–5 × length of median lobes, 1 gland spine on lateral margin of each pygidial segment; pair of setae between median lobes. Dorsal ducts smaller than marginal ducts; present in transverse linear rows; 2–5 submedial ducts present on segment 6; ca. 4 submarginal and ca. 4 submedial ducts on segment 5. Perivulvar pores: 1–3 posteromedial, 6–15 posterolateral, 12–24 posterior, 2–3 anteromedial, and 2–7 anterolateral. Trilocular pores in cluster of 1–8 near anterior spiracle; 0–3 around posterior spiracle. Microducts scattered on dorsal surface of head, plus ventral surface of abdomen. Antenna with 1 fleshy seta.

#### Comments.

*Poliaspis exocarpi* is far and away the most polyphagous and wide spread species of *Poliaspis* in Australia. There is also a considerable amount of morphological variation present among samples (e.g. the number of submedial ducts on dorsum of abdominal segment 6). The relatively small size of the median lobes (smaller than or equal in size to second lobes) and the relatively long size of the gland spines (up to 43 μm, about 5 × length of medial lobes) are also diagnostic.

**Figure 7.**
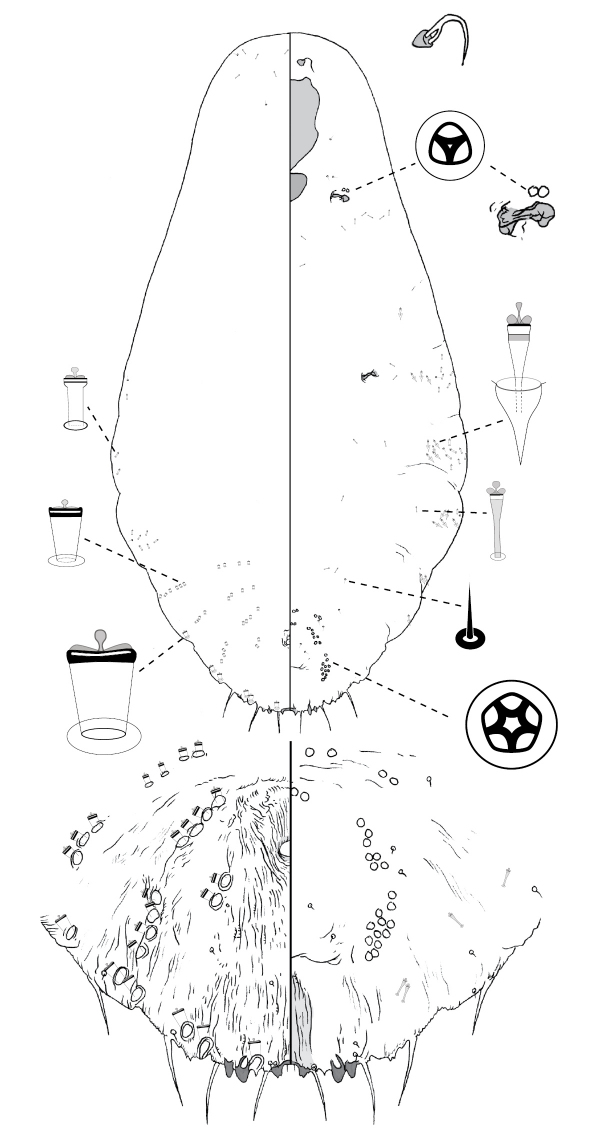
*Poliaspis exocarpi* Maskell, adult female.

### Poliaspis media

Maskell

http://species-id.net/wiki/Poliaspis_media

Fig. 8

Poliaspis media Maskell, 1880: 293
Poliaspis cycadis Comstock, 1883: 126–128. Syn. nov.
Poliaspis gaultheriae Green, 1920: 126–129. Syn. nov.

#### Material examined.

*Poliaspis media* Maskell

**Lectotype** designated by Henderson (2011). Female, New Zealand, labelled “Poliaspis media, females, from Leucopogon Fraseri (epacrid), June 1878 W.M.M.” (NZAC). This is one of 3 slides remounted from 1 original Maskell slide by RC Henderson, 2001.

*Poliaspis cycadis* Comstock

**Lectotype **female here designated, the middle female of 3 on an original slide with two labels: (a) “*Poliaspis cycadis* Comst. [= undeciphered word],” (b) “816, *Poliaspis cycadis*, C.P. & Glye,” both labels outlined in red and the coverslip ringed in black. USA: Washington DC, in conservatory, ex *Cycas revoluta* (USNM)

Paralectotypes: (i) the remaining 2 females on the lectotype slide; (ii) 1 female, same collection data (part of type material), subsequently remounted (BMNH); (iii) 2 females, each on a separate slide labelled: *Poliaspis cycadis* Comst., Type, on *Dion edulis*, Dept. Agr. D.C.; these subsequently remounted, and with scale cover under separate glass cover slip (USNM).

*Poliaspis gautheriae* Green

**Lectotype** female here designated, the female third from the label “TYPE” and marked with an arrow on the slide, in a row of 6 females: on *Gaultheria depressa*, Botanic Gardens, Edinburgh, Scotland; additional data on envelope: Coll. W. Evans, Oct 1919 (BMNH).

Paralectotypes, all with same collection data: (i) the other 5 females and 4 2nd-exuvia on the lectotype slide; (ii) 7 females, 4 2nd-exuvia and 1 1st-exuvium; (iii) 3 females; (iv) 4 females, on *Gaultheria* ‘cycadis’ (under glass), November 1919, ex coll. E.E. Green (BMNH).

#### Comments.

The type material of *Poliaspis cycadis* is morphologically inseparable from *Poliaspis media*. Both species were discovered at about the same time (1880s) and at first the host differences (cycads versus wide host range) and geographic disjunction of North America and New Zealand presented a conundrum. Examination of the type material of *Poliaspis gaultheriae*, previously synonymized with *Poliaspis cycadis* by Balachowsky (1954), revealed it to be conspecific, but the only recorded host of *Poliaspis gaultheria*e was *Gaultheria depressa*, an endemic New Zealand plant that had been transported from NZ to Scotland. Thus the logical connection to *Poliaspis media* as the senior synonym became more credible. A further point is that specimens identified as *Poliaspis cycadis* collected on *Cycas revoluta* from Kew Gardens, UK, 1887, Coll. J.W. Douglas, are misidentifications of a mixture of *Poliaspis syringae* Laing and *Furchadaspis zamiae* (Morgan). We suggest that various collections of *Poliaspis* species on cycads may be chance populations on these host plants.

**Figure 8.**
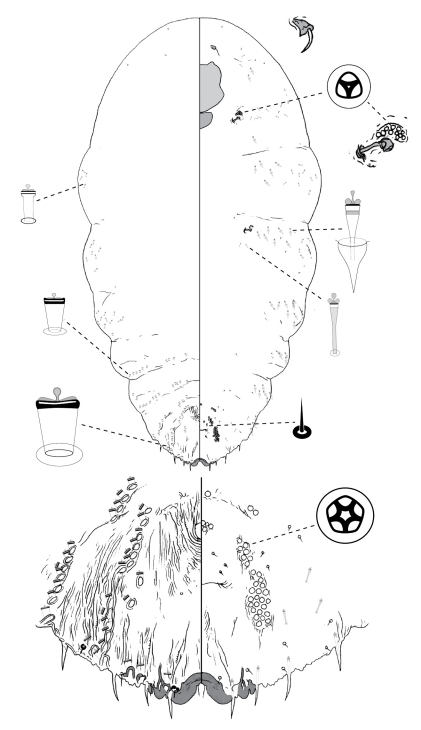
*Poliaspis media* Maskell, adult female.

### Poliaspis naamba

Hardy & Henderson sp. n.

urn:lsid:zoobank.org:act:4B995A20-0F3B-47C9-97EB-97CC3B0B52DC

http://species-id.net/wiki/Poliaspis_naamba

Fig. 9

#### Material examined.

Holotype: female: Australia, QLD, Nambour [-26.63, 152.96], of *Melaleuca* sp., 4.2.2005, C Freebaim (QDPI);

Paratypes: QLD. 2 adult females: Bray Park, Brisbane [-27.3, 152.98], ex *Melaleuca* sp., 6.3.2005, C Freebaim (QDPI); 3 adult females: Cooloola National Park [-26.1, 153.04], on leaves and stems of *Monotoca scoparia*, 7.4.1987, J Donaldson (QDPI); 3 adult females: Indooroopilly [-27.5, 152.97], on leaves of *Melaleuca* sp., 6.1989, J Grimshaw (QDPI); 2 adult females: Indooroopilly, 30.10.1998, C Neale (QDPI); 10 adult females: Indooroopilly, of *Melaleuca* sp., 28.2.2005, C Neale (QDPI); 2 adult females: Kenmore [-27.51, 152.94], on leaves of *Melaleuca nodosa*, 2.1953, G Smith (QDPI); 7 adult females: Mareeba [-16.99, 145.42], ex *Melaleuca bracteata*, 12.2004, B Pinese (QDPI); 1 adult females: Mareeba, on leaves of *Melaleuca* sp., 30.1.1982, J Donaldson (QDPI); 2 adult females: Nambour [-26.63, 152.96], of *Melaleuca* sp., 4.2.2005, C Freebaim (QDPI); 1 adult females: Yarraman [-26.84, 151.98], on leaves of *Guoia semiglauca*, 20.3.1952, F Muell. (QDPI).

#### Description, n=10.

Slide-mounted adult female 942–1475 μm long (holotype 1475 μm), body outline fusiform to pyriform, with weakly-developed lobes on pre-pygidial abdominal segments. Pygidium with 2 pairs of lobes; median lobes zygotic, divergent, lobes connected via strong sclerosis, each lobe wider than long, with rounded, dentate apex; margin between lobes incised; second lobe bi-lobed, medial lobule larger and with stronger basal sclerosis. Gland spines 19–45 μm long, 2–3 × length of median lobes, 1 gland spine on lateral margin of each pygidial segments; pair of setae between median lobes. Dorsal ducts smaller than marginal ducts; present in rows; 2–4 submedial ducts present on segment 6; ca. 4 submarginal and ca. 4 submedial ducts on segment 5. Perivulvar pores: 1–3 posteromedial, 10–15 posterolateral, 15–23 posterior, 2–4 anteromedial, and 2–5 anterolateral. Trilocular pores in cluster of 3–4 near anterior spiracle; 1–2 near posterior spiracle. Microducts numerous on dorsal surface of head, scattered on ventral surface of abdomen and thorax. Antenna with 1–2 fleshy setae.

#### Comments.

*Poliaspis naamba* is very similar to *Poliaspis waibenensis* sp. n.*Poliaspis naamba* adult females can be distinguished from those of *Poliaspis waibenensis* by (1) lacking a strong duct spur between the medial and second lobes (present in *Poliaspis waibenensis*); (2) having pores associated with the posterior spiracles (lacking in *Poliaspis waibenensis*); and (3) with pre-pygidial margin of abdomen only weakly lobed (strongly lobed in P. waibenensis). The two species also have different host associations, with *Poliaspis naamba* almost always collected from *Melaleuca* species, and *Poliaspis waibenensis* from mangrove plants.

#### Etymology.

The species name is taken from the Aborignal word *naamba* used in reference to red bottlebrush *Melaleuca viminalis*. This species has been most often found associated with *Melaleuca* species.

**Figure 9.**
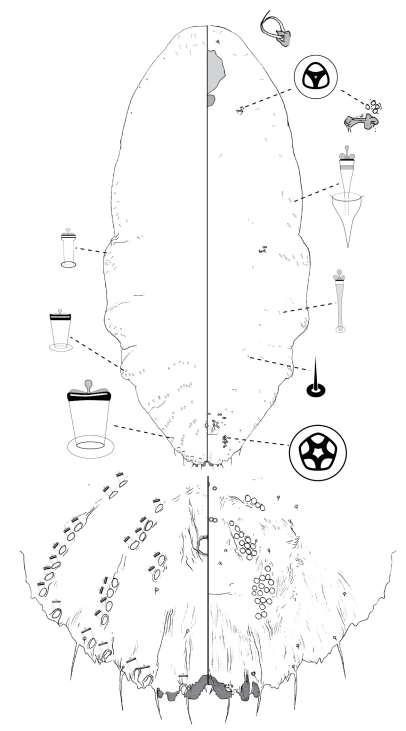
*Poliaspis naamba* Hardy and Henderson sp. n., adult female.

### Poliaspis nalbo

Hardy & Henderson sp. n.

urn:lsid:zoobank.org:act:393A0E21-FA95-4361-8242-CACB6473FEC1

http://species-id.net/wiki/Poliaspis_nalbo

Fig. 10

#### Material examined.

Holotype: female: Australia, QLD, Maleny [-26.76, 152.85], in flower heads of *Cryptandra scortechinii*, 9.1987, D Hockings (ANIC);

Paratypes: QLD. 4 adult females: same data as holotype (QDPI).

#### Description, n=5.

Slide-mounted adult female 685–998 μm long (holotype 998 μm long), body outline elongate oval, margin of thorax and pre-pygidial abdominal segments undulate. Pygidium with 2 pairs of lobes; median lobes large, zygotic, parallel, lobes connected via sclerotic strap, each lobe wider than long, rounded, with dentate apex; margin between lobes incised; second lobe not bi-lobed. Gland spines 17–24 μm long, only slightly longer than median lobes, 1 gland spine on lateral margin of pygidial segment 7 (between medial and second lobes); 2 spines on margin of segment 6; 3 spines on margin of segment 5; pair of setae between median lobes. Dorsal ducts undifferentiated from marginal ducts, except for single larger marginal duct on segment 7; present in clusters (i.e. less organized than rows); 1 submedial duct present on segment 6; ca. 7 marginal-submarginal and ca. 6 submedial ducts on segment 5. Perivulvar pores: 3–4 posteromedial, 14–16 posterolateral, 18–23 posterior, 3–6 anteromedial, and 2–5 anterolateral. Trilocular pores in cluster of 6–8 near anterior spiracle; 2–3 near posterior spiracle. Microducts absent from dorsal surface of head, scattered on ventral surface of head, thorax and abdomen. Antenna with 1 fleshy seta. Cluster of gland tubercles on ventral surface of head anterior to anterior spiracle, in addition to the submarginal / marginal clusters present more posteriorly.

#### Comments.

In contrast to many other Australian species of *Poliaspis*, which are very similar to one another, *Poliaspis nalbo* is very distinctive. It can be easily recognized by (1) the large, rounded, parallel median lobes; (2) the extra gland spines on the margin of abdominal segments 5 and 6; (3) the cluster of gland tubercles present on the ventral surface of the head (also present in *Poliaspis narungga*); (4) the absence of microducts from the dorsal surface of the head.

#### Etymology.

The species name is taken from the name of one of the aboriginal groups that originally populated the type locality Maleny, the Nalbo people.

**Figure 10.**
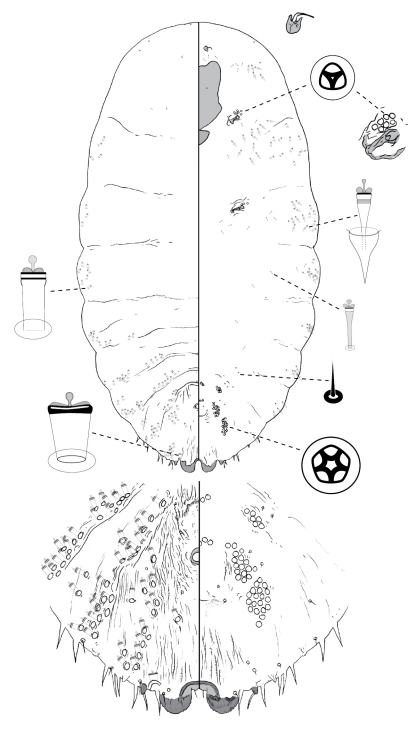
*Poliaspis nalbo* Hardy and Henderson sp. n., adult female.

### Poliaspis narungga

Hardy & Henderson sp. n.

urn:lsid:zoobank.org:act:5685916A-CDD0-448F-A4BD-D38EF9B8E7D7

http://species-id.net/wiki/Poliaspis_narungga

Fig. 11

#### Material examined.

Holotype: female: Australia, SA, Inneston, Yorke Peninsula [-35.28, 136.94], ex *Correa ?reflexa*, 1.1975, D Symon (ANIC). Holotype is on slide with 7 additional adult females arranged in two rows. Holotype is in top row, second from left.

Paratypes: SA. 7 adult females: on same slide as holotype (ANIC).

Other material:Australia, 4 adult females, intercepted in quarantine in New Zealand, Auckland, MAF, Lynfield, ex *Goeznowia vericosa*, 7.7.1997, J. McMillan, (NZAC).

#### Description, n=8.

Slide-mounted adult female 1136–1858 μm long (holotype 1552 μm long), body outline elongate oval. Pygidium with 2 pairs of lobes; median lobes zygotic, parallel, lobes connected via narrow sclerotic strap, each lobe ca. as wide as long, pointed, smaller than second lobes, margin between lobes not incised; second lobe bi-lobed, each lobule triangular, with blunt or pointed tip. Gland spines absent; pair of setae between median lobes. Dorsal ducts undifferentiated from marginal ducts; numerous on dorsum of pygidium, not organized into discrete submedial and submarginal clusters. Perivulvar pores: 2–7 posteromedial, 15–28 posterolateral, 30–50 posterior, 4–9 anteromedial, and 7–14 anterolateral. Trilocular pores in cluster of 5–8 near anterior spiracle; absent near posterior spiracle. Microducts absent from dorsal surface of head, scattered on ventral surface of head, thorax and abdomen. Antenna with 1 fleshy seta. Cluster of gland tubercles on ventral surface of head anterior to anterior spiracle, in addition to the submarginal / marginal clusters present more posteriorly.

#### Comments.

*Poliaspis narungga* is the only species of *Poliaspis* in which the adult females lack gland spines, but gland tubercles are numerous in submarginal areas of the abdomen and thorax, including a cluster anterior to the anterior spiracle. Also distinctive are (1) the lack of differentiation between marginal and dorsal ducts, and (2) the dorsal macroducts on the pygidium not being arranged into distinct submedial and submarginal clusters.

#### Etymology.

The Narungga people were the inhabitants of the Yorke Peninsula prior to the arrival of Europeans.

**Figure 11.**
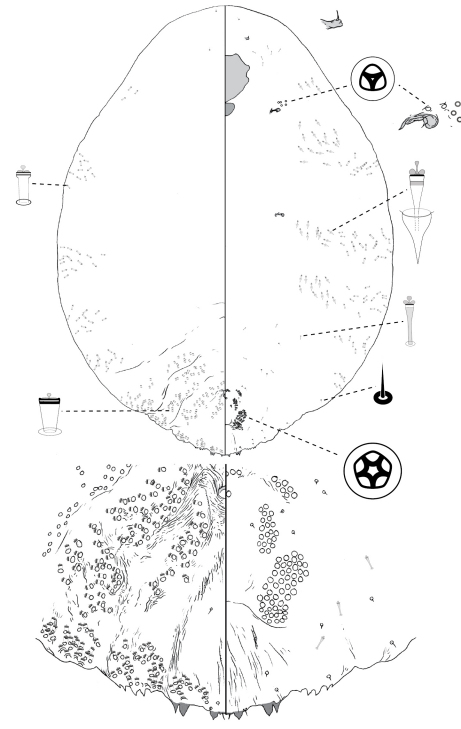
*Poliaspis narungga* Hardy and Henderson sp. n., adult female.

### Poliaspis nitens

Fuller

http://species-id.net/wiki/Poliaspis_nitens

Fig. 12

Poliaspis nitens Fuller, 1897: 5.

#### Material examined.

*Dry material*: WA. Guilford, ex *Daviesia* sp., Newman 1912, number 5, WWF 516, ASCT00006373, ASCT00006372; Kalamunda, ex *Daviesia* sp, Newman 1912, number 2, WWF 517 [corresponds to Froggatt’s collection number 516], ASCT00006371.

VIC. 2 adult females: Sandringham [-37.95, 145.00], ex *Exocarpus cupressiformis*, C French, IEE 1814; 2 adult females, same coll. data (misidentified as *Poliaspis exocarpi*) (BMNH). WA. 2 adult females: 4 miles S of Pemberton [-27.47, 153.02], ex stem of *Gastrolobium* sp*.*, 27.2.1964, SWB (QDPI). WA. 1 adult female mounted from the ASCT0000637 dry material.

VIC. 1 adult female: Dandenong Range [-37.97, 145.24], ex *Exocarpus stricta*, 2.7.1914, No. 178 G. Brittin Collection (NZAC).

#### Description, n=6.

Slide-mounted adult female 1026–1513 μm long, body outline fusiform-pyriform, thoracic and abdominal lobes not produced. Pygidium with 2 pairs of lobes; median lobes short and broad, connected medially by broad sclerosis, each lobe with rounded apex; margin between lobes not incised; second lobe bi-lobed, inner lobule with strong basal sclerosis. Gland spines 17–27 μm long, 2–5 × length of median lobes, 1 gland spine on margin of each pygidial segment; pair of setae between median lobes not observed. Dorsal ducts much smaller than marginal ducts; present in rows; 7 submedial duct present on segment 6; 6 submarginal and 7 submedial ducts on segment 5. Perivulvar pores: 1–4 posteromedial, 7–14 posterolateral, 12–26 posterior, 2–4 anteromedial, and 5–11 anterolateral. Trilocular pores in cluster of 2 near anterior spiracle; absent from posterior spiracle. Microducts scattered on head, posteromedial of anterior spiracle, anteromedial of posterior spiracle, and across abdomen. Antenna with 1 long, curved fleshy setae.

#### Comments.

Fuller (1897) described the median lobes of *Poliaspis nitens* as being very short and wide. That is unique among *Poliaspis* species and matches the material we have examined from ASCU, which was also collected from the same host and area. No setae were observed between the median lobes, but there appear to be a pair of empty setal sockets present and it is possible that the setae have broken off.

The adult female of *Poliaspis nitens* can be distinguished from other species of *Poliaspis* on the basis of the very short and broad median lobes. Three other species treated here have median lobes smaller than the second lobes: *Poliaspis callitris*, *Poliaspis exocarpi* and *Poliaspis araucariae*. In *Poliaspis exocarpi* and *Poliaspis araucariae* the body margin between median lobes is slightly incised, and in *Poliaspis araucariae* the median lobes are strongly divergent. In *Poliaspis callitris* the body margin is not clearly incised between the median lobes, but each medial lobe is longer than wide and has a pointed apex.

**Figure 12.**
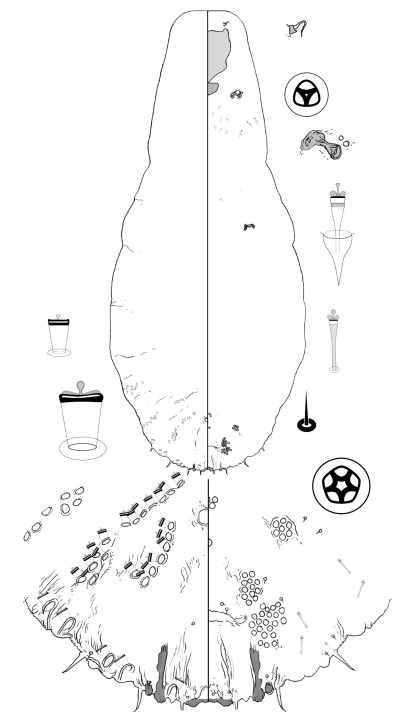
*Poliaspis nitens* Fuller, adult female.

### Poliaspis ozothamnae

Hardy & Henderson sp. n.

urn:lsid:zoobank.org:act:1E486663-2B38-4C1A-A2B7-225A5062FA40

http://species-id.net/wiki/Poliaspis_ozothamnae

Fig. 13

#### Material examined.

Holotype: female: Australia, QLD, Brisbane [-27.47, 153.02], ex *Ozothamnus diosmifolius*, 17.4.1986, N Gough (ANIC).

Paratypes: QLD. 6 adult females: same data as holotype (QDPI). SA. 7 adult females: Second Valley [-35.52, 138.22], ex *Pulteneae involucrata*, 13.10.1965, HM Brookes (ANIC).

#### Description, n=10.

Slide-mounted adult female 809–1256 μm long, body outline turbinate. Pygidium with 2 pairs of lobes; median lobes zygotic, parallel, close-set, lobes connected via narrow sclerosis, each lobe wider than long, apex obtuse-rounded and dentate; margin between lobes not incised; second lobe not bi-lobed, roughly pointed, apex notched in some specimens, close to medial lobe. Gland spines 8–17 μm long, about as long as median lobes, gland spine absent from margin of pygidial segment 7 (between medial and second lobes), 4–7 spines on margin of each of abdominal segments 5–6; pair of setae between median lobes. Marginal ducts: 1 on abdominal segment 7, 1 on segment 6, not differentiated from dorsal ducts on segment 5. Dorsal ducts present in clusters (i.e. several ducts across); ca. 5 submedial ducts present on segment 6; ca. 12 marginal-submarginal and ca. 12 submedial ducts on segment 5. Perivulvar pores: 3–6 posteromedial, 7–15 posterolateral, 10–18 posterior, 4–5 anteromedial, and 3–6 anterolateral. Trilocular pores in cluster of 7–16 near anterior spiracle; 2–8 near posterior spiracle. Microducts absent on dorsal surface of head, scattered on ventral surface of thorax and abdomen. Antenna with 1 fleshy seta. Gland tubercles absent from ventral surface of head anterior to anterior spiracle.

#### Comments.

*Poliaspis ozothamnae* is distinguishable from other species of *Poliaspis* by having (1) the second lobe close set to medial lobe, without a gland spine near lateral edge of medial lobe; (2) only 2 differentiated marginal ducts; (3) the 2–8 pores near each posterior spiracle; and (4) having multiple gland spines on each pygidial segment other than 8.

#### Etymology.

The species name is taken from the host name *Ozothamnus diosmifolius*.

**Figure 13.**
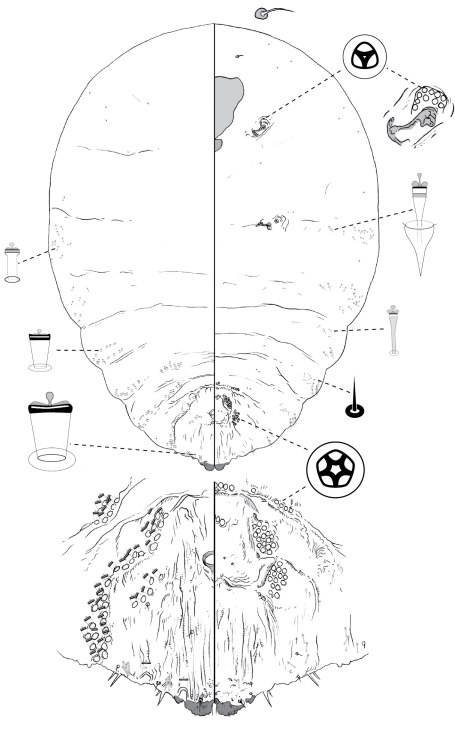
*Poliaspis ozothamnae* Hardy and Henderson sp. n., adult female.

### Poliaspis syringae

Laing

http://species-id.net/wiki/Poliaspis_syringae

Fig. 14

Poliaspis syringae Laing, 1929: 17–19.

#### Material examined.

**Lectotype** female here designated: Australia, Victoria, Kew, on lilac, C Plumridge, No. 40 (1925), L.B.E. 1413 (BMNH).

Paralectotypes: collection data same as lecototype: 3 females on one slide.

Other material: QLD. 4 adult females: 12 Mile Barramundi Reserve, near Bajool [-23.65, 150.64], ex native citrus, 28.9.2005, R Elder (QDPI); 5 adult females: Darnley Island [-9.58, 143.79], ex *Capparis* sp., 25.4.1996, J Grimshaw, JFG 3291 (QDPI); 4 adult females: Hannaford [-27.44, 150.04], ex *Eremocitrus glauca*, 4.7.1979, P Fayden (QDPI);13 adult females: Marmor [-23.68, 150.71], ex *Eremocitrus glauca*, 10.1955, A Brimblecombe (QDPI); 2 adult females: Yarraman [-26.84, 151.98], ex *Capparis nobilis*, 8.1947, A Brimblecombe, 2111; 1314/10729 (QDPI); 2 females, UK, Royal Gardens, Kew, England, ex coll. J.W. Douglas, on *Cycas revoluta*, 1887 (BMNH).

#### Description, n=10.

Slide-mounted adult female 453–1662 μm long, body outline oval, margin of thorax and abdomen undulate. Pygidium with 2 pairs of lobes; median lobes large, zygotic, divergent, connected via a narrow strap, each lobe wider than long, apex rounded and dentate; margin between lobes incised; second lobe bi-lobed, lateral lobule minute, each lobule with rounded apex, basal scleroses absent. Gland spines 17–34 μm long, 1–2 × as long as median lobes, 1 gland spine on margin of each pygidial segment; pair of setae between median lobes. Dorsal ducts smaller than marginal ducts, in rows; ca. 4 submedial ducts present on segment 6; ca. 6 submarginal and ca. 3 submedial ducts on segment 5. Perivulvar pores: 2–6 posteromedial, 10–15 posterolateral, 13–24 posterior, 1–6 anteromedial, and 2–4 anterolateral. Trilocular pores in cluster of 5–19 near anterior spiracle; 1–3 near posterior spiracle. Microducts absent on dorsal surface of head, scattered on ventral surface of thorax and abdomen. Antenna with 1 fleshy seta.

#### Comments.

*Poliaspis syringae* is most similar to *Poliaspis naamba* and *Poliaspis waibenensis*. Adult females of *Poliaspis syringae* can be distinguished from those by (1) median lobes much larger than second lobes (similar in size in *Poliaspis naamba* and *Poliaspis waibenensis*); and (2) second lobes without basal sclerosis (present in P. *naamba* and P. *waibenensis*). The specimens examined here are from *Eremocitrus* (or native citrus) and *Capparis*. The specimens from *Capparis* are larger than those from *Eremocitrus* (smallest from *Capparis* 1233 μm, largest from *Eremocitrus* 1056 μm), have more pores around the anterior spiracle (ca 18 vs ca. 6), and have slightly longer gland spines on the longer side of the range observed among samples from *Eremocritus*.

**Figure 14.**
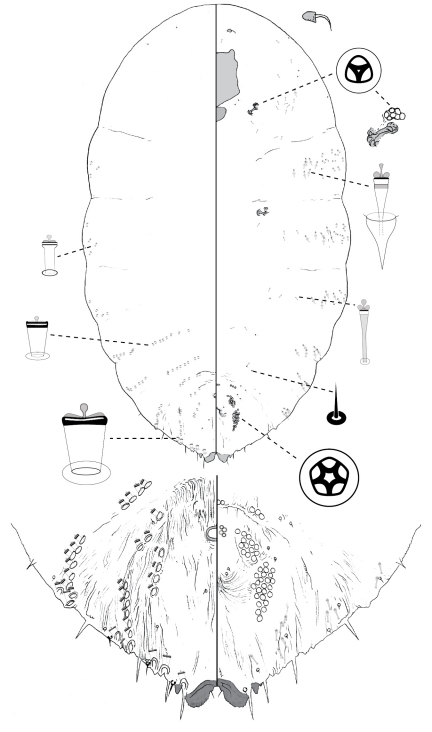
*Poliaspis syringae* Laing, adult female.

### Poliaspis waibenensis

Hardy & Henderson sp. n.

urn:lsid:zoobank.org:act:4A9E74E1-428A-4257-9FCE-65A2FDDC1AE1

http://species-id.net/wiki/Poliaspis_waibenensis

Fig. 15

#### Material examined.

Holotype: female: Australia, QLD, Thursday Island [-10.58, 142.22], on leaves of *Lumnitzera racemosa*, 2.9.2004, B Waterhouse (ANIC).

Paratypes: QLD. 10 adult females: Atherton [-17.27, 145.48], on leaves of *Parsonsia straminea*, 1.2.1982, J Donaldson (QDPI); 6 adult females: Hammond Island [-10.55, 142.21], on leaves of *Rhizophora* sp., 29.11.1993, J Grimshaw (QDPI); 7 adult females: Thursday Island, on leaves of *Pemphis acidula*, 6.9.1983, J Donaldson (QDPI); 5 adult females: Thursday Island, ex mangrove, 16.5.1985, J Donaldson (QDPI); 4 adult females: same data as holotype (QDPI). WA. 3 adult females: Sunday Island [-16.4, 123.19], on leaves of *Ficus* sp., 13.5.2002, A Williams (QDPI); 3 adult females: Willie Creek via Broome [-17.96, 122.24], ex mangrove, 7.8.2003, A Postle, C Brockway (QDPI).

#### Description, n=10.

Slide-mounted adult female 1101–2040 μm long (holotype 2040 μm long), body outline fusiform to pyriform, with weakly-developed lobes on pre-pygidial abdominal segments. Pygidium with 2 pairs of lobes; median lobes zygotic, divergent, lobes connected via broad (more than half width of lobes) sclerosis, each lobe wider than long, with rounded apex; margin between lobes incised; second lobe bi-lobed, ca. as large as medial lobe, medial lobule larger and with stronger basal sclerosis. Gland spines 25–47 long long, 2–3 × length of median lobes, 1 gland spine on lateral margin of each pygidial segment; pair of setae between median lobes. Conspicuous, duct spur present between medial and second lobe, as long as medial lobe. Dorsal ducts smaller than marginal ducts; present in rows; 4 submedial ducts present on segment 6; ca. 6 submarginal and ca. 6 submedial ducts on segment 5. Perivulvar pores: 2–6 posteromedial, 6–11 posterolateral, 15–28 posterior, 0–3 anteromedial, and 4–7 anterolateral. Trilocular pores in cluster of 1–5 near anterior spiracle; absent near posterior spiracle in most specimens (2 present in specimens from *Parsonsia*). Microducts absent on dorsal surface of head, scattered on ventral surface of abdomen and thorax. Antenna with 2 fleshy setae.

#### Comments.

*Poliaspis waibenensis* is very similar to *Poliaspis naamba*. See comments under *Poliaspis naamba* for discussion.

#### Etymology.

The species name is taken from the Torres Strait Islander name for Thursday Island: Waiben, meaning ‘place of no water.’

**Figure 15.**
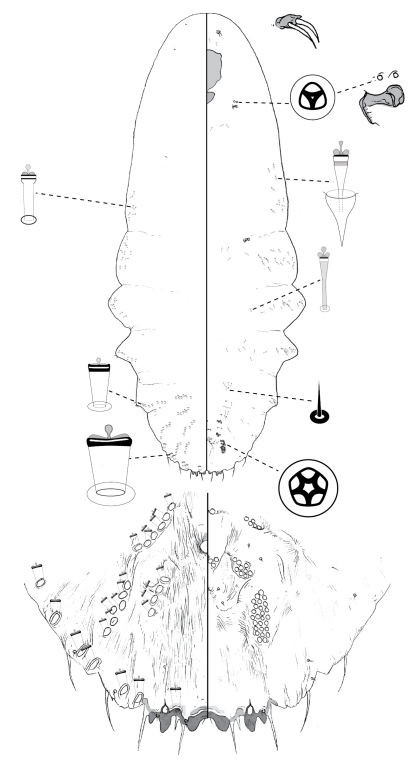
*Poliaspis waibenensis* Hardy and Henderson sp. n., adult female.

### Poliaspis wilga

(Leonardi) comb. n.

http://species-id.net/wiki/Poliaspis_wilga

Fig. 16

Mytilaspis wilga Leonardi, 1903: 43–44.
Lepidosaphes wilga (Leonardi), change of combination, Sanders, 1906: 17.
Leonardaspis wilga (Leonardi), change of combination, MacGillivray, 1921: 287.

#### Material examined.

Lectotypefemale here designated, female slide labelled: *‘*on wilga, *Geijera parviflora*, Condobolin, 17.x.1900, WW Froggatt (339), BM 1964–4, CIE’ (BMNH).

Paralectotypes: 12 slides with same data as Lectotype (BMNH); 1 slide with 4 females labelled “co-type, *Mytilaspis wilga*, Green [crossed out and replaced in pencil by ‘Leon.’], ‘Wilga’ *Geijera parviflora*, NS Wales, Australia, coll. WW Froggett no. 339 [no date].” On cover: “*Lepidosaphes wilga* Leon., on *Geijera*, Australia, coll. WW Froggatt, BM 1940, 180, [no collection date].”

Other Material: NSW. 2 adult female: Glenmore via Bourke [-30.09, 145.94], ex *Eremophila* sp., 10.3.1994, D Sparks (QDPI); 5 adult females: McCatheys, Dunsandle Rds [? ], on leaves of *Eremophila deserti*, 11.10.1994 (QDPI). VIC. 6 adult females: Merbein [-34.17, 142.05], ex *Myoporum* sp., 8.3.1948, P26 (QDPI).

#### Description, n=10.

Slide-mounted adult female 939–1542 μm long (Holotype 1130 μm long), body outline oval. Pygidium with 1 pair of lobes; median lobes small, zygotic, parallel, lobes connected via narrow sclerosis, each lobe wider than long, rounded; margin between lobes not incised. Gland spines 9–24 μm long, ca. 1–2 × as long as median lobes, 1 gland spine on lateral margin of each pygidial segment; pair of setae between median lobes. Marginal ducts: 1 on abdominal segment 7, ca. 4 on segment 6, ca. 3 on segment 5. Dorsal ducts smaller than marginal ducts; present in clusters (i.e. less organized than rows); 4–6 submedial ducts present on segment 6; 5–7 marginal-submarginal and ca. 9 submedial ducts on segment 5. Perivulvar pores: 3–4 posteromedial, 10–19 posterolateral, 13–27 posterior, 0–4 anteromedial, and 1–8 anterolateral. Trilocular pores in cluster of 2–5 near anterior spiracle; absent near posterior spiracle. Microducts numerous on dorsal surface of head, scattered on ventral surface of thorax and abdomen. Antenna with 2 fleshy setae.

#### Comments.

*Poliaspis wilga* is the only species of *Poliaspis* to have only the median lobes present. It can also be recognized by the loose groupings of more than 2 marginal ducts on abdominal segments 6 and 7.

**Figure 16.**
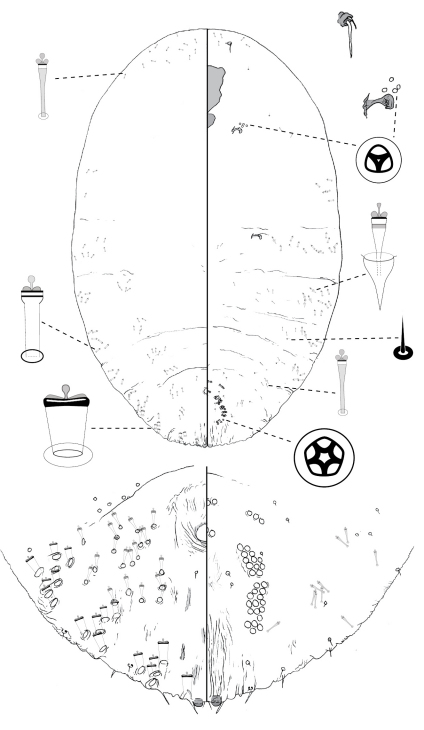
*Poliaspis wilga* (Leonardi) comb. n., adult female.

### Laingaspis lanigera

(Laing)

http://species-id.net/wiki/Laingaspis_lanigera

Fig. 17

Poliaspis lanigera Laing, 1929: 20–21.
Laingaspis lanigera (Laing), Borchsenius and Williams 1963: 365.

#### Material examined.

Lectotypefemale here designated, adult female on slide labelled: Australia, NT, Darwin, on mangrove, GF Hill, BM 1916–225 [with “Type” label] (BMNH).

Paralectotypes: adult female on slide with same data as Lectotype, marked as Paratype [sic] (BMNH); 4 slides of females with same basic data but variously with “Port Darwin on Foreshore, IBE 647,” “GF Hill no. 682, from type material, BM 1940–180,” “IBE 151 / GE Hill 682” (BMNH); 6 poor quality single-specimen slide mounts of males, labelled “Australia, Port Darwin, GF Hill, 16.vi.1916, BM 1922–155” – probably part of the type series even though rigistered years later.

Other material: QLD. 5 adult females: Jacky Jacky Creek [-10.94, 142.51], on leaves of *Aegiceras corniculatum*, 16.1.1998, J Grimshaw, B Waterhouse (QDPI); 5 adult females: New Mapoon [-10.87, 142.39], on stems of *Acalypha milkeriana*, 14.1.1998, J Grimshaw (QDPI).

#### Description.

Slide-mounted adult female 965–1126 μm long (Holotype 1026 μm long), body outline oval. Margin of pygidium rounded, serrate. Median lobes dinstinct, with pointed apices, rest of pygidial margin densely-packed with gland spines and sclerotic teeth, some of which may be homologous to lobes. Anus in anterior third of pygidium; opening round. Dorsomedial ducts on pygidium longer than marginal and submarginal ducts; present in 2 clusters near anus; ca. 5 ducts in anterior cluster and ca. 3 in posterior one. Trilocular pores in cluster of 2–3 around each anterior spiracle, absent near posterior spiracles. Antenna with 2 fleshy setae. Perivulvar pores quinquelocular, in 7–8 groups; 5 groups on abdominal segment 7, and 2 or 3 groups on abdominal segment 6; 0–4 posteromedial, 5–7 posterolateral, 11–15 posterior, 0–3 anteromedial, and 0–4 anterolateral. Dorsal ducts 1-barred; dense along margin and submargin of pygidial dorsum, plus a few in clusters near anus. Gland tubercles in marginal / submarginal clusters on thoracic and pre-pygidial abdominal segments.

#### Comments.

This species has the diagnostic distribution of perivulvar pores found among species of *Poliaspis* but differs in an important feature: namely, 1-barred ducts arranged in a dense marginal swath on the pygidial dorsum. 1-barred ducts and gland tubercles, which are also present in this species, is an unusual combination among armored scale insect species.

**Figure 17.**
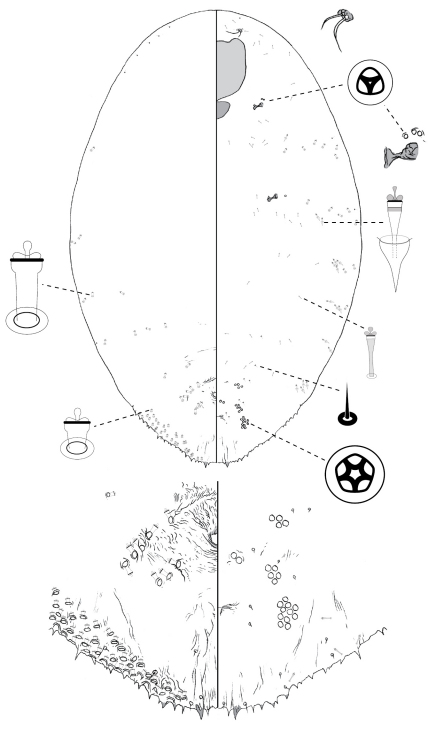
*Laingaspis lanigera* (Laing), adult female.

## Supplementary Material

XML Treatment for Poliaspis

XML Treatment for Poliaspis alluvia

XML Treatment for Poliaspis araucariae

XML Treatment for Poliaspis attenuata

XML Treatment for Poliapsis callitris

XML Treatment for Poliaspis ceraflora

XML Treatment for Poliaspis elongata

XML Treatment for Poliaspis exocarpi

XML Treatment for Poliaspis media

XML Treatment for Poliaspis naamba

XML Treatment for Poliaspis nalbo

XML Treatment for Poliaspis narungga

XML Treatment for Poliaspis nitens

XML Treatment for Poliaspis ozothamnae

XML Treatment for Poliaspis syringae

XML Treatment for Poliaspis waibenensis

XML Treatment for Poliaspis wilga

XML Treatment for Laingaspis lanigera
